# Genetic Parameter Estimation for Pregnancy Loss and Their Association With Reproductive and Growth Traits in Brahman Cattle Under Extensive Tropical Conditions

**DOI:** 10.1111/jbg.70025

**Published:** 2025-11-03

**Authors:** Daniel Cardona‐Cifuentes, Lucia G. de Albuquerque, Milagros Arias, Sindy Caivio‐Nasner, Luis Camaripano, Luis G. Gonzalez‐Herrera, Patricia I. Schmidt, Fernando Baldi

**Affiliations:** ^1^ Departamento de Zootecnia Faculdade de Ciências Agrarias e Veterinárias, Universidade Estadual Paulista (UNESP), Via de Acesso Professor Paulo Donato Castellane Jaboticabal São Paulo Brazil; ^2^ Facultad de Ciencias Agrarias, Fundación Universitaria Agraria de Colombia – UNIAGRARIA Bogotá Colombia; ^3^ Conselho Nacional de Desenvolvimento Científico e Tecnológico (CNPq) Brasilia DF Brazil; ^4^ Facultad de Ciencias Veterinarias, Universidad Central de Venezuela Maracay Venezuela; ^5^ Departamento Técnico Baspel LTDA Santa Cruz de la Sierra Bolivia; ^6^ Grupo de Investigación en Biodiversidad y Genética Molecular (BIOGEM) Universidad Nacional de Colombia Sede Medellín Medellín Colombia; ^7^ Grupo de Melhoramento Animal e Biotecnologia (GMAB), Faculdade de Zootecnia e Engenharia de Alimentos, Departamento de Medicina Veterinária Universidade de São Paulo (USP) Pirassununga São Paulo Brazil

**Keywords:** animal breeding, beef cattle, genetic correlation, genomic selection, single‐step GBLUP

## Abstract

This study estimated genetic parameters for pregnancy loss (PL) in Brahman cattle and evaluated the genetic correlation of PL with growth and reproductive traits using both the pedigree relationship matrix (**A**) and pedigree plus genomic relationship matrix (**H**). Data were collected from two herds in Bolivia, focusing on three age groups: heifers, primiparous and multiparous cows. Threshold animal models were fitted to each group. Multitrait models were fitted between the PL at different age group and between PL and the following traits: adjusted weights at 450 (W450) and 550 (W550) days, scrotal circumference adjusted at 450 (SC450) and 550 (SC550) days, accumulated cow productivity (ACP), age at first calving (AFC) and stayability (STAY). The **H** matrix increased the heritability for PL in heifers from 0.06 to 0.11. The genetic correlation between PL in heifers and primiparous cows changed using H (from 0.18 to 0.7), and it was high between heifers and multiparous cows. Moderate‐high negative genetic correlation was observed between PL and STAY, with changes in heifers when using **H** (−0.17 to −0.57). AFC and PL presented medium‐high positive genetic correlations. Negative correlations between PL and SC450 or SC550 were found in primiparous and multiparous cows. Using **H**, the correlation between PL in heifers and ACP shifted from 0.08 to −0.31, showing medium‐high negative correlations for the other two age groups. Genetic correlations were low between PL and W450 or W550. Genomic information allows the use of PL as a selection criterion in heifers. Selection for major sexual precocity, longevity and productivity would enable the reduction of pregnancy loss.

## 1 Introduction

Pregnancy loss (PL) is defined as early embryonic or late fetal mortality (Reese et al. 2020) and is one of the most significant issues causing economic losses in tropical beef cattle operations (Pohler et al. 2020). Some noninfectious factors associated with PL in *B. indicus* are genetics and inbreeding (maternal and paternal), maternal factors (age and parity order), hormonal protocols for reproductive biotechnology applications, nutritional balance, previous postpartum problems (retained placenta, metritis, endometritis), heat stress, among others (Consentini et al. 2023; Diskin et al. 2016). According to Prado et al. (2024), most of the pregnancy losses in *B. indicus* occurs in fetal mortality period between 60 and 150 days of gestation, showing that heifer pregnancy losses can duplicate losses in mature cows.

Some scarce studies of genetic parameters for gestational loss in dairy cattle have reported that the trait's heritability varied between 2% and 18% (Ask‐Gullstrand et al. 2021; Sigdel et al. 2022). However, the moderate‐to‐low heritability estimate for PL is not a constraint to include this trait in beef cattle breeding programmes (Diskin et al. 2016). Quantifying the genetic variability for pregnancy loss is necessary to assess the importance of using this trait as a selection criterion in *B. indicus* beef cattle. Additionally, it is important to explore the correlation between reproductive traits such as pregnancy loss and traditional traits evaluated in beef cattle breeding programmes to explore possible favourable and unfavourable associations.

The single‐step GBLUP (ssGBLUP) (Misztal et al. 2009) has earned great interest in commercial breeding programmes since it allows the combination of pedigree and phenotypic information from genotyped and nongenotyped individuals using all the available information. The ssGBLUP has been implemented in Nellore and Brahman cattle for estimating variance components, enabling more accurate inference of genetic parameters (Cardona‐Cifuentes et al. 2024; Raidan et al. 2018). Nevertheless, authors have reported that including genomic information can yield biased variance components due to the genotyping of only selected subsets of animals (Gao et al. 2019; Wang et al. 2020).

Using the ssGBLUP approach is promising for understanding PL genetic variation and its association with traditional production traits in beef. Nevertheless, it must be verified how much the estimates differ from those obtained using the traditional pedigree‐based relationship matrix, and which approach allows for better modelling and lower estimation error. Then, this work aims to estimate variance components and genetic parameters for pregnancy loss in Brahman cattle and to assess the genetic correlation of pregnancy loss with growth and reproductive traits evaluated in a commercial breeding programme, comparing the use of the numerator relationship matrix (**A**) and the additive and genomic relationship matrix (**H**) from the ssGBLUP.

## 2 Material and Methods

### 2.1 Phenotypic and Genotypic Information

Pregnancy loss records were collected for Brahman cattle raised in two herds in Bolivia, Estancias Espiritu and San Judas, located in Beni and Santa Cruz states. The weaning happened between 6 and 8 months of age, and the animals were reared within pasture‐grazing systems with mineral supplementation.

Both herds have sanitary programmes to prevent reproductive diseases and protect the fetus during gestation, mainly during the 60‐ to 150‐day period. A vaccine against bovine viral diarrhoea, infectious bovine rhinotracheitis and Leptospira is applied in females at the beginning of the breeding season, with a booster dose 20–30 days later and a third dose at moment of rectal palpation for pregnant cows. Sires receive an annual dose 15–30 days prior to the start of the breeding season. For brucellosis, strain 19 vaccine is administered to females aged 3–8 months, and annual serological tests are carried out on the males for brucellosis detection. Heifers enter the breeding season at an average age of 2 years and a minimum weight of 280 kg.

The breeding season lasts 90–100 days between October and January. Artificial insemination is used for 45–60 days. Cows that fail to achieve pregnancy through artificial insemination are moved to controlled natural mating with multiple clean‐up bulls. When it is observed that the cows do not return to oestrus, pregnancy is confirmed through rectal palpation around 60 days after insemination or breeding season. Cows that are confirmed pregnant but are observed to have signs of abortion or did not give birth are recorded as pregnancy loss. Then, cows with a successful pregnancy are assigned a phenotype of 1, and cows with pregnancy loss are assigned a phenotype of 2.

**FIGURE 1:**
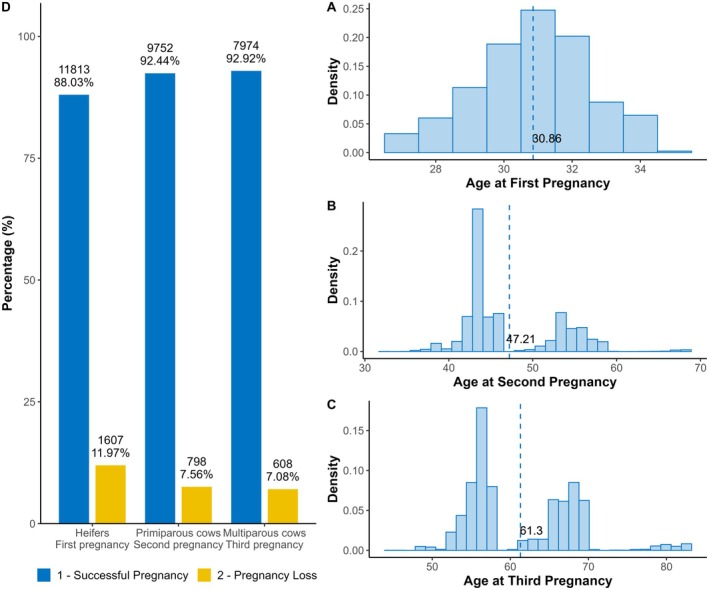
Distribution of age (months) at first (A), second (B), and third (C) pregnancies, and percentage of successes and losses by pregnancy order (D). [Colour figure can be viewed at wileyonlinelibrary.com]

Records of mating seasons between 1998 and 2021 were collected for cows in three pregnancy orders: nulliparous heifers (first pregnancy), primiparous cows (second pregnancy) and lactating multiparous cows (third pregnancy). Moreover, breeders apply selection by culling all females that present pregnancy loss as heifers or as primiparous cows. Figure 1 shows the number of records, the proportion of pregnancy successes and loss and the age distribution at pregnancy in months in each pregnancy order. The heifer, primiparous and multiparous cows were the progeny of 886, 473 and 152 sires, respectively.

Pregnancy loss records were correlated with growth and reproductive traits collected in the Brahman Breeding Program of the National Association of Breeders and Researchers (ANCP, Ribeirão Preto, SP). These traits are weights adjusted to 450 and 550 days of age (W450 and W550), scrotal circumference adjusted to 450 and 550 days of age (SC450 and SC550, respectively), age at first calving (AFC), accumulated cow productivity (ACP) and Stayability (STAY). The ACP is a trait expressed in kilograms of calf weaned per year and is calculated according to (Grossi et al. 2008)

$$\mathrm{ACP}=\frac{\mathrm{WW}\times n_{c}\times 365}{{\mathrm{ADC}}_{n}-550}$$

where WW is the average weaning weight of the dam's calves adjusted for 210 days of age, $n_{c}$ is the total number of calves, and ${\mathrm{ADC}}_{n}$ is the age of the dam in days at last calving, 365 is a constant corresponding to an annual production basis, and 550 refers to the first calving goal at 30 months, with a minimum breeding age of 18 months. For STAY, cows with at least three calvings up to 76 months of age had a phenotype categorised as success (2) or failure (1) otherwise. Table 1 Shows the descriptive statistics.

**TABLE 1:** Descriptive statistics and number of animals with phenotypic records (*N*), for growth and reproductive traits in Brahman cattle.

Trait	Mean ± SD	*N*	Heifers		Primiparous cows		Multiparous cows	
			SPR	CBR	SPR	CBR	SPR	CBR
W450 (kg)	234.4 ± 28.78	11,616	177	2012	157	875	146	326
W550 (kg)	278.9 ± 34.18	38,409	271	11,548	260	8690	243	6496
SC450 (cm)	21.4 ± 2.55	5844	157	—	141	—	131	—
SC550 (cm)	24.9 ± 3.18	19,065	264	—	253	—	237	—
ACP (kg/year)	137.2 ± 22.94	9575	258	8341	258	8949	244	7744
AFC (months)	35.7 ± 2.54	13,346	286	11,847	268	10,429	244	7871
STAY (%)^a^	60.57	4438	239	3944	238	3602	231	2845

Abbreviations: ACP, accumulated cow productivity; AFC, age at first calving; CBR, cows with both records (PL and any growth or reproductive trait); *N*, number of animals with phenotypic records; SC450, scrotal circumference adjusted at 450 days of age; SC550, scrotal circumference adjusted at 550 days of age; SD, standard deviation; SPR, Number of sires with progeny records; W450, Adjusted weight at 450 days of age; W550, Adjusted weight at 50 days of age.

^a^ Represents percentage of success for STAY.

Animals were genotyped with a moderate‐density panel with approximately 50,000 SNP markers (GGP Indicus 50 K Neogen). The PREGSF90 within the GIBBSF90+ programme was used to perform genomic quality control (Lourenco et al. 2022). Animals with call rates lower than 0.9 or Mendelian conflict higher than 0.01 were removed, as well as monomorphic markers, markers with call rates lower than 0.9, minor allele frequency lower than 0.05, mendelian conflict higher than 0.2 and those located on sexual chromosomes. After quality control, 39,521 effective SNP markers were considered for 3436 animals. Of these animals, 886, 473 and 152 were heifers, primiparous and multiparous with records, respectively.

### 2.2 Genetic Parameter Estimation

Variance components and genetic parameters for pregnancy loss were estimated using a single‐trait threshold animal models that considered the pedigree information (**A** matrix) and pedigree plus genomic information through the **H** matrix from the single‐step GBLUP (ssGBLUP) approach (Aguilar et al. 2010). The inverse of **H** matrix (**H** ^−1^) can be assembled as follows, with **G** ^−1^ being the inverse of the genomic relationship matrix as in VanRaden (2008) and $A_{22}^{-1}$ the inverse of A for genotyped animals.

$$H^{-1}=A^{-1}+\left[\begin{array}{cc} 0 & 0\\ 0 & G^{-1}-A_{22}^{-1} \end{array}\right]$$

Generalised linear models were fitted using the *glm* procedure of R software environment (R Core Team 2024) with a *logit* function to find the model that better explained PL probability. Likelihood‐ratio tests, AIC and BIC were used to select the best model. For PL, contemporary groups were formed by herd, year and month of rectal palpation. Initially, these models ignored the relationship between animal random effects; when considered, the Deviance Information Criterion (DIC) was used to choose the best model. For W450, W550, SC450 and SC550, the contemporary group was composed of farm, year, birth season (dry season from April to September and rainy season from October to March), sex and management group (animals raised in the same paddock). For AFC, ACP and STAY, the CG was composed of farm, year and birth season.

Linear animal models were fitted for W450, W550, SC450, SC550, ACP and AFC, whereas threshold animal models were used for PL and STAY. The PL was analysed using single‐trait models for each pregnancy order (age group) and multitrait models were fitted to evaluate the relationships between PL at different age groups. Moreover, multitrait models were fitted between PL in each pregnancy order and the other productive and reproductive traits. The general model was as follows: y=Xb+Zg+Wc+e, where ***y*** is a vector of dependent variables (W450, W550, SC450, SC550, AFC, ACP or underlying liability for STAY and PL), ***b*** is a vector of fixed effects (linear covariable of age at pregnancy for PL and CG for the other traits), ***g*** is the vector of animal additive genetics effects, ***c*** is the vector for random effect of contemporary group only included for PL, and ***e*** is a vector for random residual effects. ***X***, ***Z*** and ***W*** are incidence matrices that relate ***y*** with ***b***, ***g*** and ***c***, respectively.

The random effects were assumed to be normally distributed with mean zero and $\mathrm{Var}\left(c\right)=I\sigma_{c}^{2}$ for contemporary group random effect, $\mathrm{Var}\left(g\right)=A\sigma_{g}^{2}$ or $\mathrm{Var}\left(g\right)=H\sigma_{g}^{2}$ for direct additive genetic effects. Where, **A** is the numerator relationship matrix, **H** is the pedigree plus genomic relationship matrix and I an identity matrix, $\sigma_{g}^{2}$ is the additive genetics variance, and $\sigma_{c}^{2}$ is the variance of contemporary group effect. In the case of two‐trait models the assumptions followed: $\mathrm{Var}\left(g\right)=A⊗S_{g}$ or $\mathrm{Var}\left(g\right)=H⊗S_{g}$; $\mathrm{Var}\left(c\right)=I⊗S_{c}$; $\mathrm{Var}\left(e\right)=I⊗S_{e}$. Where $S_{g}=\left[\begin{array}{cc} \sigma_{g1}^{2} & \sigma_{g12}\\ \sigma_{g21} & \sigma_{g2}^{2} \end{array}\right]$ is the genetic variance and covariance matrix; $S_{c}=\left[\begin{array}{cc} \sigma_{c1}^{2} & \sigma_{c12}\\ \sigma_{c21} & \sigma_{c2}^{2} \end{array}\right]$ is the variance and covariance matrix for PL contemporary group random effect with $\sigma_{c12}=0$ and $\sigma_{c2}^{2}=0$, and $S_{e}=\left[\begin{array}{cc} \sigma_{e1}^{2} & \sigma_{e12}\\ \sigma_{e21} & \sigma_{e2}^{2} \end{array}\right]$ the residual variance and covariance matrix. For binary traits, contemporary groups with less than five animals a without both phenotypic levels were removed. Records higher or lower than 3.5 standard deviations from the CG mean, or CG with less than four animals were removed for the linear traits.

According to the threshold animal model the (co)variance components and genetic parameters for PL and STAY were estimated assuming an underlying scale with normal distribution (Mrode 2005): $u|\theta \sim N\left(W\theta ,I\sigma_{e}^{2}\right)$, where, **u** is the vector of the base scale with order *r* (number of animals); $\theta^{\prime }=\left(b^{\prime },g^{\prime },c^{\prime }\right)$ is the parameters vector with order *s* (*s*: number of effects in the model); **W** is the incidence matrix with order *r* by *s*; **I** is the identity matrix with order *r* by *r*; and $\sigma_{e}^{2}$ is the residual variance. For threshold models, the residual variance is fixed in $\sigma_{e}^{2}=1$ (Sorensen and Gianola 2002). The single‐trait and two‐trait analysis were performed using Bayesian inference by the Gibbs sampling algorithm implemented in GIBBSF90+ software (Lourenco et al. 2022). Gibbs chains of 1,000,000 iterations were generated with burn‐in of 200,000 and a sampling interval of 10 cycles. The posterior means for covariance components were calculated using 80,000 samples; heritability and genetic correlations were calculated in each iteration, and subsequently, the mean of samples was estimated. The convergence was tested using visual inspection and Geweke test (Geweke 1992) implemented by the Bayesian Output Analysis (BOA) package (Smith 2007) in R Core Team (2024).

## 3 Results

Pregnancy loss was 12%, 7.6% and 7% for heifers, primiparous and multiparous cows, respectively. The heritability estimated for PL was low in the three pregnancy orders, as expected for reproductive traits with strong environmental influence or complex modelling (Table 2). For pregnancy loss in heifers, genomic information (**H** Matrix) increased the heritability estimates and allowed better modelling (lower DIC).

**TABLE 2:** Posterior mean, HPD and DIC for pregnancy loss heritability in Brahman cattle, using a pedigree–relationship matrix (**A** matrix) and pedigree–genomic relationship matrix (**H** matrix).

	**A** matrix			**H** matrix		
	Mean (SD)^a^	HPD	DIC	Mean (SD)^a^	HPD	DIC
Heifers	0.06 (0.02)	0.02–0.11	−25901.9	0.11 (0.04)	0.04–0.19	−28192.6
Primiparous cows	0.07 (0.03)	0.02–0.13	−31466.7	0.08 (0.04)	0.02–0.15	−31810.9
Multiparous cows	0.09 (0.04)	0.02–0.18	−28077.2	0.09 (0.05)	0.02–018	−28059.4

Abbreviations: DIC, deviance information criterion; HPD, highest posterior density intervals.

^a^ Posterior standard deviation in brackets.

Using the **H** matrix significantly changed the genetic correlation estimates between PL in heifers and primiparous cows since the genetic correlation changed from low to high with the inclusion of genomic information. The genetic correlation for pregnancy loss was consistently high and positive in all other cases (Table 3). Moreover, using the **H** matrix allowed a reduction in DIC for the model and a smaller posterior standard deviation for genetic correlations between PL in different pregnancy orders.

**TABLE 3:** Posterior mean, HPD and DIC for genetic correlation of pregnancy loss in different pregnancy order in Brahman cattle, using pedigree–relationship matrix (**A** matrix) and pedigree–genomic relationship matrix (**H** matrix).

	**A** matrix			**H** matrix		
	Mean (SD)^a^	HPD	DIC	Mean (SD)^a^	HPD	DIC
Heifers and Primiparous cows	0.18 (0.34)	−0.37 to 0.97	−73253.1	0.70 (0.23)	0.26–0.99	−94094.8
Heifers and Multiparous cows	0.66 (0.30)	0.07–0.99		0.63 (0.26)	0.15–0.98	
Primiparous and Multiparous cows	0.62 (0.23)	0.18–0.98		0.74 (0.21)	0.32–0.99	

Abbreviations: DIC, deviance information criterion; HPD, highest posterior density intervals.

^a^ Posterior standard deviation in brackets.

**FIGURE 2:**
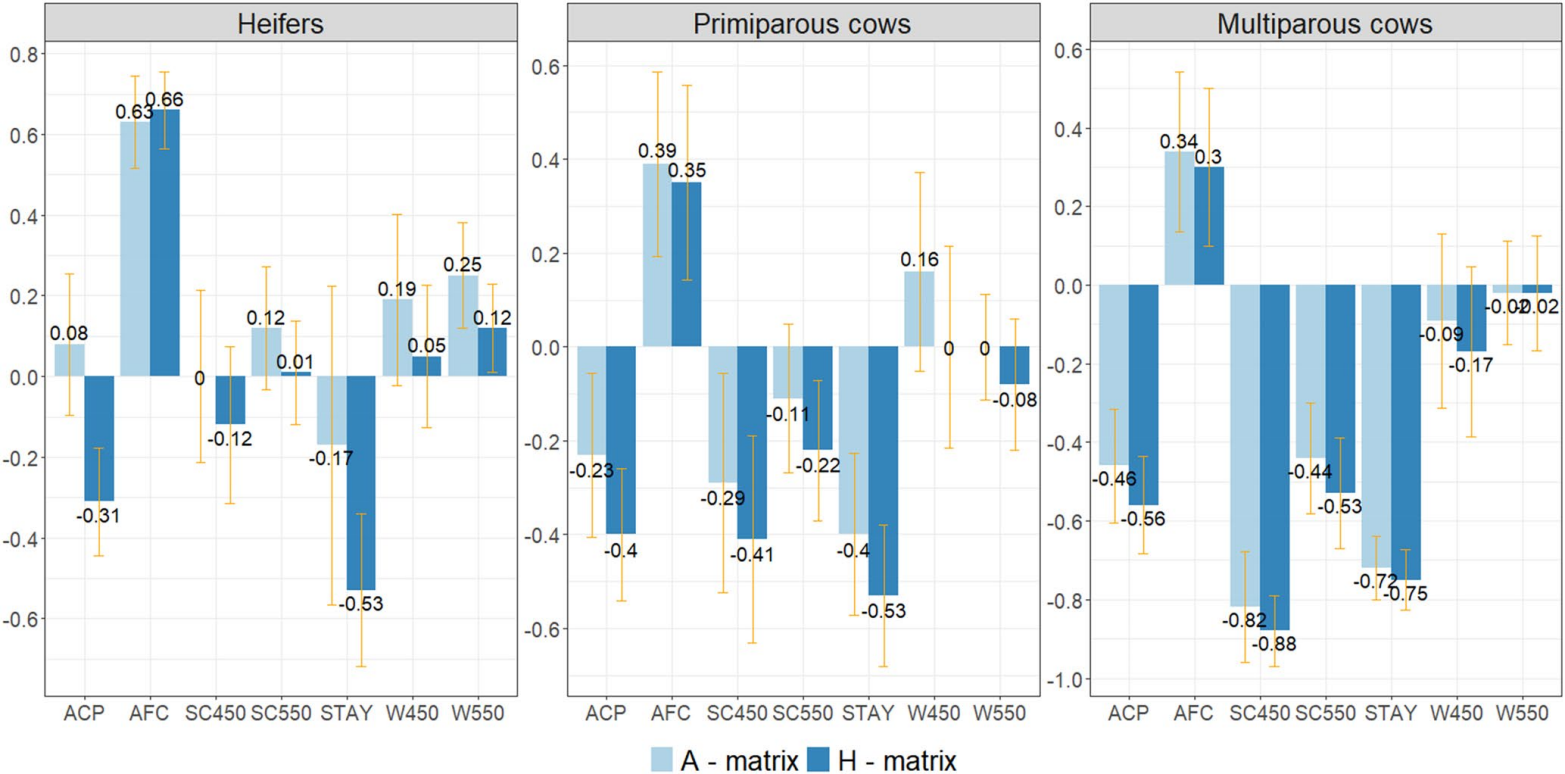
Posterior means and standard deviations for genetic correlations between pregnancy loss and productive and reproductive traits in Brahman cattle, estimated using the pedigree‐based relationship matrix (**A** matrix) and the combined pedigree–genomic relationship matrix (**H** matrix). Traits include adjusted weights at 450 (W450) and 550 (W550) days, scrotal circumference at 450 (SC450) and 550 (SC550) days, accumulated cow productivity (ACP), age at first calving (AFC) and stayability (STAY). [Colour figure can be viewed at wileyonlinelibrary.com]

Generally, the genetic correlations between pregnancy loss and production and reproduction traits maintain the same direction. However, they may change magnitude with the use of the **H** matrix. In general, considering genomic information allows for reducing the posterior standard deviation (Figure 2) and the magnitude of HPD of genetic correlation estimates, as well as the DIC of the models (Data S1).

The AFC and PL showed medium‐to‐high and positive genetic correlations, with the highest values observed for heifers. In this case, estimates using the **H** or **A** matrix were similar. A low genetic correlation was observed between scrotal circumference (SC450 and SC550) and PL in heifers and primiparous cows, except for the moderate negative value for SC450 and PL when using the **H** matrix in primiparous cows. The PL in the multiparous cows and scrotal circumference presented a moderate‐to‐high negative genetic correlation.

Using genomic information increases the magnitude of the negative genetic correlation between PL in heifers and STAY. The genetic correlation between PL and STAY was negative and moderate to high for primiparous and multiparous cows. The genetic correlation between pregnancy loss and ACP in the heifers showed an important change since it went from 0.08 (no association) when using the **A** matrix to −0.31 (negative and low) when using the **H** matrix. In the other cases, a low to medium negative genetic correlation was observed between pregnancy loss and ACP. Using the **H** matrix, genetic correlation estimates between PL and STAY or between PL and ACP showed a relatively small posterior standard deviation and HPD interval. However, DIC was smaller using the **A** matrix (Data S1).

On the other hand, using the traditional relationship matrix displayed a low positive genetic correlation between PL (in all three groups) and the weights adjusted for 450 and 550 days. Using the **H** matrix, the genetic correlation estimates between PL (all three pregnancy orders) and weight adjusted to 450 and 550 days were close to zero, which means little change with both pedigree matrices.

## 4 Discussion

Studies for Nellore cattle have reported that pregnancy loss is higher in heifers than in cows and higher in primiparous than in multiparous cows (Consentini et al. 2023; Prado et al. 2024). There are no reports of genetic parameters for pregnancy loss in *B. indicus* cattle, but some studies on dairy cattle also found low heritability for this trait. Ask‐Gullstrand et al. (2021) reported a heritability of 7% for pregnancy loss derived from progesterone milk measures for Swedish Red and Swedish Holstein. Carthy et al. (2016, 2015) used a threshold model in a multiracial dairy cattle population and estimated heritability of 0.02 for PL in models that also include heteroses and permanent environmental effects. Bamber et al. (2009) reported a heritability of 0.16 for US Holstein cattle in threshold models considering the sire–maternal grandsire effect.

Using the **A** matrix, heritability for PL was higher in multiparous cows than in heifers, which is consistent with results from Sigdel et al. (2022) for Holstein cattle (2% for nulliparous heifers, 8% for primiparous cows, and 18% for multiparous cows) when considering fetal loss as a binary trait and analysing using **A**‐based threshold models.

Using the **H** matrix increased the heritability of PL in heifers, surpassing the values observed in later ages. For some authors, this implies the possibility of reducing PL by incorporating this trait into dairy cattle breeding programmes (Diskin et al. 2016), as it can be as heritable as traditional fertility traits (Ask‐Gullstrand et al. 2021; Sigdel et al. 2022). Likewise, Data S2 and reports from Cavani et al. (2015) indicate that heritability estimates for pregnancy loss in Brahman cattle are not significantly different from those for other reproductive traits, such as STAY and AFC. Then, pregnancy loss could be a trait included in selection programmes for Brahman cattle, particularly if the selection focuses on heifers, since the PL in the first pregnancy is a phenotype measured early, with a greater number of records, sires and genotypes available.

However, the heritability increase displayed by the **H** matrix may reflect inflated genetic variance estimates, as shown in broilers (real and simulated data) when using the **H** matrix under selective genotyping (Wang et al. 2020). Pregnancy loss is a trait with high selection pressure and selective genotyping, as most of the genotyped animals exhibit pregnancy success (phenotype equal to 2), and due to the culling of females with failure.

The **G** matrix (VanRaden 2008), included in **H**, may overestimate genomic relationships, since it is sensitive to allele frequencies and can confuse recent relatedness with distant population structure (García‐Baccino et al. 2017; Conomos et al. 2016). That could bias the variance component estimation; therefore, it is essential to have adequate compatibility between pedigree and genomic information, as well as the inclusion of genotyped animals with phenotypes (Misztal et al. 2021).

**FIGURE 3:**
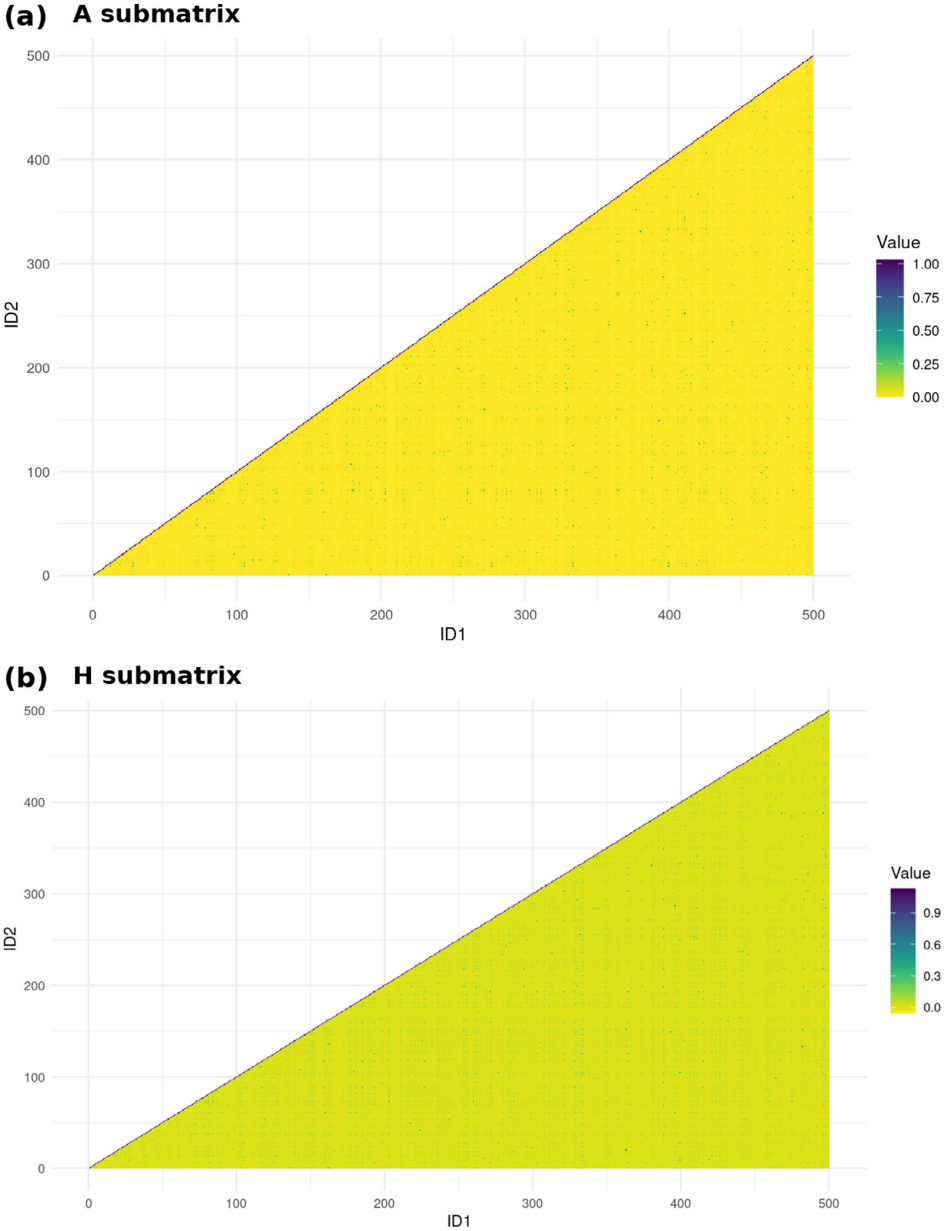
Genetic relationship structure for the first 500 animals in the pedigree: (a) using pedigree‐based relationships (A submatrix) and (b) combined pedigree and genomic relationships (H submatrix). [Colour figure can be viewed at wileyonlinelibrary.com]

Legarra et al. (2009) showed that the **H** matrix transmits genomic information to nongenotyped animals, thereby increasing the value of relationship coefficients. Figure 3 illustrates the genetic relationship structure for a subsample of 500 individuals. The yellow areas in Figure 3a represent null relationships among unrelated individuals based solely on pedigree, whereas Figure 3b reveals higher genetic relationship coefficients when genomic information is included. Moreover, Data S3 shows that both herds presented a Pedigree Completeness Index close to zero, since 7479 animals (55% of animals with records) had at least one unknown parent (Maccluer et al. 1983). This is due to the use of multi‐sire herds in extensive management of the indicine cattle, where it is impossible to determine the sire of future dams. Therefore, the **H** matrix facilitates the recovery of genetic relationship information that would be difficult to capture using the **A** matrix.

For Aguilar et al. (2010), the augmented relationship information may result in higher additive variance. This explains the increased heritability observed for PL in heifers, as it is the age with the most phenotype and pedigree records, leading to a greater influence of the **H** matrix. In Nellore cattle, reports have shown that estimating variance components using the **H** matrix leads to an increase in knowledge of relationships between individuals, resulting in higher heritability estimates, even when there are gaps in the pedigree (Tonussi et al. 2021).

Considering only the pedigree relationships displayed low genetic correlation estimates for PL in heifers and primiparous cows. That is consistent with Sigdel et al. (2022), who observed low genetic correlations (0.22 to 0.31) between fetal loss across possible parities, so the author suggested that different pregnancies should be considered different traits.

In these results, using the **H** matrix increased the genetic correlation between heifers and primiparous cows, as well as between primiparous and multiparous cows. Therefore, the high positive genetic correlation observed between PL in different pregnancy orders means that in Brahman cattle, heifers with a low probability of PL will maintain this behaviour as mature cows. In datasets for multitrait models, the culling of heifers that experienced pregnancy loss resulted in missing phenotypic records for the second trait (gestation as primiparous cows). A similar pattern may occur in the transition from primiparous to multiparous cows.

As seen in Figure 3, the **H** matrix extends the genomic relationship to no genotyped animals (Legarra et al. 2009). Then, the **H** matrix increased the accounted genetic covariation structure, thereby increasing the genetic correlation between PL in heifers and primiparous cows. The extension of genomic relationships also explains why the **H** matrix displayed genetic correlation estimates of greater magnitude (more negatives) between PL and other reproductive traits. In Nelore Cattle, Gordo et al. (2016) observed that the **H** matrix captured a higher proportion of genetic covariance, leading to increased genetic correlations between carcass traits and visual scores.

The **H** matrix improved the estimation of genetic correlation between PL at different pregnancy orders, and between PL and reproductive traits, as indicated by the lower posterior standard deviations and narrower HPD Intervals. Moreover, the increase in heritability for PL in the first pregnancy was also associated with a lower DIC, as well as multi‐trait models between PL and scrotal circumference (SC450 and SC550) or between PL and AFC. That implies a better model fit compared with the **A** matrix. Therefore, using the **H** matrix showed advantages for estimating genetic parameters.

Other reports have shown that using the **H** matrix allows for reducing the standard error of genetic parameter estimates (Aldridge et al. 2020; Gordo et al. 2016). Cardona‐Cifuentes et al. (2024) argue that the use of the **H** matrix enables a better consideration of selection steps, thereby improving variance component estimation for traits under intense selection pressure or selective genotyping in Nelore cattle.

In the present Brahman cattle population, genetic correlation near zero for PL with W450 and W550 is a favourable situation since it is evidence that the selection to increase the growth rate of animals would not affect the PL. Genetic correlation estimates between ACP and PL in heifers exhibited a change in direction with the **H** matrix approach. Thus, it becomes more biologically reasonable since heifers with pregnancy loss will not become highly productive cows. At the same time, this favourable negative genetic correlation between PL and ACP implies that the selection for more productive cows would allow for heifers and cows with a small probability of pregnancy loss.

The estimated genetic correlation between STAY and PL was expected to be negative because a cow that loses one of its first three pregnancies may be culled or fail to achieve a successful phenotype in STAY. Selecting heifers with a lower probability of pregnancy loss would allow cows to stay longer in the herd; this is relevant since it increases beef cattle profitability for the producer thanks to the reduction of replacement rate due to a higher proportion of longer‐lived cows (da Silva Neto et al. 2020).

Cows that reach higher ages at first parity exhibit reduced sexual precocity and fertility; these animals are more likely to experience pregnancy loss. That corresponds with the high positive genetic correlation observed for AFC and PL. Then, a favourable correlated response in PL is expected when selecting for sexual precocity, reducing pregnancy loss in the first three pregnancies. STAY and AFC are traits with a negative favourable genetic correlation in zebu breeds (Cavani et al. 2015; Kluska et al. 2021). Therefore, the selection to increase sexual precocity in heifers also increases the productivity of beef cattle operations; it allows the cows to begin generating profits at earlier ages, increasing their longevity and leaving more calves for the herd (Beretta et al. 2001; Kluska et al. 2021).

This finding suggests that selecting sires with higher scrotal circumference could lead to cows with a lower probability of pregnancy loss, which may have important practical implications since in zebu cattle scrotal circumference is a reproductive trait with moderate heritability (Data S2) and high selection intensity, that is used as a criterion to select males with better fertility and more precocious daughters (da Silva Neto et al. 2020; Terakado et al. 2021). Moreover, reports have shown an association between sire fertility and pregnancy loss due to the importance of paternal genetics in pregnancy maintenance (Franco et al. 2020, 2018).

Genomic information is a promising tool for establishing the relationship between pregnancy loss and other cattle reproductive traits, while allows a more robust selection of sires and dams with lower probability of pregnancy losses, since this tool has increased the accuracy of breeding values for low heritability traits in zebu cattle (Fernandes Júnior et al. 2022; Kluska et al. 2018).

## 5 Conclusion

Pregnancy loss is a promising selection criterion to include in zebu breeding programmes under tropical conditions, and simultaneous selection to reduce PL in heifers and mature cows is possible. Selection for growth traits is not related to pregnancy loss. Selecting sires and cows for major sexual precocity, longevity and productivity would allow for the reduction of pregnancy loss. Moreover, selecting males with greater scrotal circumference and more precocious heifers would reduce pregnancy losses. The use of the **H** matrix may overestimate the genetic (co)variance structure; nevertheless, in this Brahman cattle population under extensive management and with pedigree gaps, it enabled a reduction in the posterior standard deviation and HPD intervals of genetic parameter estimates and better model adjustment, thereby improving the estimation of heritability and genetic correlations.

## Ethics Statement

Ethical approval was not necessary for this research because no animals were submitted to experimental conditions; the data came from regular reproductive records collected for commercial herds.

## Conflicts of Interest

The authors declare no conflicts of interest.

## Supporting information


**Data S1:** jbg70025‐sup‐0001‐DataS1.pdf.

**Data S2:** jbg70025‐sup‐0002‐DataS2.pdf.

**Data S3:** jbg70025‐sup‐0003‐DataS3.pdf.

## Data Availability

The data that support the findings of this study are available from the corresponding author upon reasonable request.
