# Supplementary material for: Genetic Parameter Estimation for Pregnancy Loss and Their Association With Reproductive and Growth Traits in Brahman Cattle Under Extensive Tropical Conditions
Source: J Anim Breed Genet. 2025 Nov 3;143(2):365–74. doi: 10.1111/jbg.70025 (PMC12887145; doi:10.1111/jbg.70025)
Supplement: Supplementary file 1 — Data S1: jbg70025‐sup‐0001‐DataS1.pdf. [file JBG-143-365-s003.pdf]

Supplementary material 1

Highest posterior density (HPD) intervals and deviance information criterion (DIC) for genetic correlations between pregnancy loss and productive and reproductive traits in Brahman cattle, using the pedigree relationship matrix (A matrix) and the pedigree-genomic relationship matrix (H matrix).

		**A** matrix			**H** matrix		
		HPD			HPD		
[a]Trait	Pregnancy order	Min.	Max.	DIC	Min.	Max.	[2]DIC
SC450	Heifers	-0.42	0.40	56202.53	-0.50	0.26	53654.71
SC450	Primiparous cows	-0.72	0.17	41219.12	-0.80	0.02	40235.39
SC450	Multiparous cows	-0.99	-0.53	37469.00	-0.98	-0.70	36855.69
SC550	Heifers	-0.18	0.41	148424.34	-0.25	0.25	-579326.56
SC550	Primiparous cows	-0.42	0.20	133228.36	-0.51	0.08	-595017.19
SC550	Multiparous cows	-0.71	-0.17	129973.83	-0.80	-0.26	-685394.39
AFC	Heifers	0.40	0.83	-311476.44	0.47	0.84	-334225.39
AFC	Primiparous cows	0.01	0.74	13651.09	-0.05	0.73	13305.58
AFC	Multiparous cows	-0.06	0.71	28126.64	-0.09	0.69	27911.67
W450	Heifers	-0.22	0.61	186033.13	-0.29	0.39	183225.28
W450	Primiparous cows	-0.26	0.57	169508.55	-0.41	0.43	167372.53
W450	Multiparous cows	-0.50	0.35	164143.59	-0.59	0.28	163564.90
W550	Heifers	-0.01	0.48	382499.84	-0.10	0.33	383041.46
W550	Primiparous cows	-0.21	0.23	389931.49	-0.35	0.20	389653.32
W550	Multiparous cows	-0.28	0.24	403643.09	-0.31	0.26	404481.34
ACP	Heifers	-0.25	0.42	36555.35	-0.57	-0.04	42098.78
ACP	Primiparous cows	-0.57	0.11	-85309.27	-0.67	-0.12	-83736.77
ACP	Multiparous cows	-0.74	-0.18	22451.13	-0.80	-0.31	23980.66
STAY	Heifers	-0.92	0.66	-675223.57	-0.89	-0.17	-648213.99
STAY	Primiparous cows	-0.71	-0.05	-1437678.66	-0.81	-0.23	-1028079.33
STAY	Multiparous cows	-0.86	-0.55	-2392287.72	-0.89	-0.60	-1422247.03

W450 = Adjusted weight at 450 days of age; W550 = Adjusted weight at 450 days of age; SC450 = scrotal circumference adjusted at 450 days of age; SC550 = scrotal circumference adjusted at 550 days of age, AFC = age at first calving, ACP = accumulated cow productivity. Min = Minimum values for highest posterior density interval; Max = Maximum values for highest posterior density interval.

[1] Green color highlights smaller DIC values for **H** matrix
