# Supplementary material for: Genetic Parameter Estimation for Pregnancy Loss and Their Association With Reproductive and Growth Traits in Brahman Cattle Under Extensive Tropical Conditions
Source: J Anim Breed Genet. 2025 Nov 3;143(2):365–74. doi: 10.1111/jbg.70025 (PMC12887145; doi:10.1111/jbg.70025)
Supplement: Supplementary file 2 — Data S2: jbg70025‐sup‐0002‐DataS2.pdf. [file JBG-143-365-s001.pdf]

Supplementary material 2

Heritability estimates and 95% highest posterior density (HPD) intervals for production and reproduction traits in Brahman cattle, using the pedigree relationship matrix (A matrix) and the pedigree-genomic relationship matrix (H matrix).

	**A** Matrix		**H** Matrix	
Trait	Heritability	HPD	Heritability	HPD
SC450	0.34	0.25-0.42	0.35	0.27-0.44
SC550	0.45	0.40-0.49	0.44	0.40-0.49
W450	0.35	0.29-0.42	0.37	0.31-0.43
W550	0.32	0.30-0.34	0.32	0.29-0.34
AFC	0.10	0.07-0.13	0.10	0.07-0.14
APC	0.16	0.1-0.21	0.21	0.17-0.25
STAY[1]	0.10	0.04-0.17	0.14	0.08-0.21

W450 = Adjusted weight at 450 days of age; W550 = Adjusted weight at 450 days of age; SC450 = scrotal circumference adjusted at 450 days of age; SC550 = scrotal circumference adjusted at 550 days of age, AFC = age at first calving, ACP = accumulated cow productivity.
