# Supplementary material for: Genetic Parameter Estimation for Pregnancy Loss and Their Association With Reproductive and Growth Traits in Brahman Cattle Under Extensive Tropical Conditions
Source: J Anim Breed Genet. 2025 Nov 3;143(2):365–74. doi: 10.1111/jbg.70025 (PMC12887145; doi:10.1111/jbg.70025)
Supplement: Supplementary file 3 — Data S3: jbg70025‐sup‐0003‐DataS3.pdf. [file JBG-143-365-s002.pdf]

Supplementary material 3

Descriptive statistics for the Pedigree Completeness Index in the two evaluated herds

Herd	Mean	SD	Minimum	Maximum	Generations	n
SJ	0.09	0.22	7.69E-11	1	30	412
EE	0.08	0.23	1.72E-12	1	30	13008

SJ = San Judas; EE = Estancia Espíritu, SD= Standard deviation; n = animals with records. Pedigree Completeness Index computed according to Maccluer et al. (1983) using optiSel package (https://cran.r-project.org/web/packages/optiSel/index.html) in R cran project.
